# Extending the Lipidome Coverage by Combining Different Mass Spectrometric Platforms: An Innovative Strategy to Answer Chemical Food Safety Issues

**DOI:** 10.3390/foods10061218

**Published:** 2021-05-28

**Authors:** Jérémy Marchand, Yann Guitton, Estelle Martineau, Anne-Lise Royer, David Balgoma, Bruno Le Bizec, Patrick Giraudeau, Gaud Dervilly

**Affiliations:** 1 LABERCA, Oniris, INRAE, 44307 Nantes, France; jeremy.marchand@hotmail.fr (J.M.); yann.guitton@oniris-nantes.fr (Y.G.); anne-lise.royer@oniris-nantes.fr (A.-L.R.); david.balgoma@ilk.uu.se (D.B.); bruno.lebizec@oniris-nantes.fr (B.L.B.); 2 CEISAM UMR 6230, Université de Nantes, CNRS, 44000 Nantes, France; estelle.martineau@univ-nantes.fr; 3 SpectroMaîtrise, CAPACITES SAS, 26 Bd Vincent Gâche, 44200 Nantes, France

**Keywords:** serum, lipidomics, Lipidyzer™, LC-HRMS, ractopamine, β-agonist

## Abstract

From a general public health perspective, a strategy combining non-targeted and targeted lipidomics MS-based approaches is proposed to identify disrupted patterns in serum lipidome upon growth promoter treatment in pigs. Evaluating the relative contributions of the platforms involved, the study aims at investigating the potential of innovative analytical approaches to highlight potential chemical food safety threats. Serum samples collected during an animal experiment involving control and treated pigs, whose food had been supplemented with ractopamine, were extracted and characterised using three MS strategies: Non-targeted RP LC-HRMS; the targeted Lipidyzer™ platform (differential ion mobility associated with shotgun lipidomics) and a homemade LC-HRMS triglyceride platform. The strategy enabled highlighting specific lipid profile patterns involving various lipid classes, mainly in relation to cholesterol esters, sphingomyelins, lactosylceramide, phosphatidylcholines and triglycerides. Thanks to the combination of non-targeted and targeted MS approaches, various compartments of the pig serum lipidome could be explored, including commonly characterised lipids (Lipidyzer™), triglyceride isomers (Triglyceride platform) and unique lipid features (non-targeted LC-HRMS). Thanks to their respective characteristics, the complementarity of the three tools could be demonstrated for public health purposes, with enhanced coverage, level of characterization and applicability.

## 1. Introduction

While the use of anabolic compounds has been banned in livestock for more than 30 years [1], the recently updated regulatory scheme confirms such provision [2]. From a public health perspective related to the chemical safety of food from animal origin, it reaffirms the European commitment to the performance of the associated controls. In this firmly reaffirmed context, the search for ever more innovative control strategies is even more topical. In particular, continuing and extending the promising work initiated 15 years ago on the investigation of the physiological effects induced as a consequence of illegal practices through metabolomics approaches appears to be a priority [3,4]. Although main proofs of concepts have been obtained focusing on the polar metabolome [5,6,7,8,9,10,11], the apolar and lipidic fraction was also shown to be relevant while highlighting the disruption of phosphatidylglycerols (PG), phosphatidylethanolamine (PE), phosphatidylcholine (PC) and phosphatidic acid (PA) in bovine serum upon trenbolone/estradiol administration [12] and the disruption of PE, phosphatidylinositol (PI) and sphingomyelin (SM) in muscle tissues collected on ractopamine (RAC) fed pigs [13]. However, these preliminary results did not allow a thorough characterisation of the lipidome since the non-targeted methods applied lacked proper data validation or extensive lipid coverage. Moreover, because the application of these forbidden veterinary drugs in livestock aims at modifying the animal carcass composition for leaner meat promotion, a significant shift of associated lipid profiles is expected, justifying further methodological efforts to be dedicated to allowing robust lipidomics to be applied. Characterising lipidome disruptions as a consequence of growth promoter application would indeed allow generating new knowledge on the mechanism of action of these anabolic agents and especially discovering relevant biomarkers for more efficient screening of such practices.

Lipidomics, which has grown as a major field over the last decade, is recognised as a complex compartment of the metabolome [14,15,16,17] with many sample preparation methods [18,19,20,21] and mass spectrometric-based analysis methods [22,23,24,25,26,27,28]. Across these numerous methods, two main categories can be distinguished and referred to as targeted approaches—where a limited number of specific lipid classes or species are monitored and quantified—and non-targeted approaches—where an open-list of compounds is analysed for subsequent identification using annotation tools [29]. The latter provides rich information on the lipidome, as they theoretically allow the measurement of any detectable lipid signals [30], resulting in thousands of features. However, this requires data cleaning steps to remove noise and redundancies (isotopes, adducts). Moreover, the assignment of these signals remains a challenging step of the workflow. In contrast, targeted approaches are more selective, thus increasing confidence in the results, even if the acquired information is much more limited. Globally, across the diverse methods and strategies, it appears that no single workflow is sufficient for a wide and complete lipidome characterisation. In such a context, the combination of non-targeted and targeted approaches from various complementary techniques is expected to provide an optimal strategy [31] that would further allow discovering unexpected biomarker signals.

The present article describes the implementation of three MS platforms (namely: non-targeted LC-HRMS, Lipidyzer™ and an in-house platform for triglyceride regioisomers) to determine changes in lipidomic profiles in serum of ractopamine treated pigs. Since they differ in technology (ion mobility, LC, HRMS, MS/MS) and approach (targeted, non-targeted), this combination is expected to provide both enhanced lipid coverage and reliability in the obtained results. In comparison with other multi-platform approaches published by other groups [31], this original strategy aims to further enhance TG analysis, using a dedicated platform for quantifying their regioisomeric composition.

## 2. Materials and Methods

### 2.1. Animal Experiment

The blood samples used in this study were obtained from a previously described ethically approved experiment [32], specifically designed to evaluate the disruptions induced in pig blood serum metabolite profiles upon ractopamine administration. Two groups constituted of randomly divided 5 healthy 4-month-old female pigs, involved over 4 weeks. After a 3-day acclimatisation, animals from the treated group were exposed to RAC hydrochloride (Sigma Aldrich, Saint Quentin Fallavier, France) through a 10 ppm daily dose in pre-weighted feed (corresponding to 0.45 mg/kg bw/day). The dosage for each animal was verified through complete eating of the daily portion. A total of 6 blood samples were collected, respectively at Day-3 (D3), Day-9 (D9), Day-16 (D16), Day-18 (D18), Day-23 (D23) and Day-29 (D29) for each individual from both groups: control (individuals P1 to P5) and treated (individuals P6 to P10). The samples were then allowed to clot at room temperature in order to obtain serum samples.

QC samples were prepared by pooling the same amount of all collected and carefully homogenised samples.

All samples were prepared into suitable 100 µL aliquots and immediately stored at −20 °C until analysis.

### 2.2. Analytical Platforms

To characterise the lipidome as widely as possible and evaluate the added value of combining multiple tools, 3 mass spectrometry platforms were involved for the analysis of the samples from the animal experiment, each of them providing a different level of characterisation. A first option was the non-targeted analysis using Reversed-Phase Ultra High-Performance Liquid Chromatography coupled to mass spectrometry (RP UHPLC-HRMS), completed by the targeted platform Lipidyzer™ (differential ion mobility associated with shotgun lipidomics), dedicated to the quantitative analysis of lipids from several classes and finally an in-house developed LC-HRMS platform able to quantify the regioisomeric composition of triglycerides (TG).

For each platform, a dedicated sample preparation protocol was carried out, as described in Table 1. While the non-targeted approach was applied on all samples, the targeted tools were implemented for samples collected at the beginning and end of the animal experiment, based on the results from the former. Each time, specific parameters and processing were used, as well as quality assurance (QA) and quality control procedures (QC), which are summarised in Table 1. The full details of the sample preparation and analysis procedures can be found in Supplementary Materials.

### 2.3. Data Analysis

For non-targeted data, multivariate analysis was performed using SIMCA 13.0.2 (Umetrics AB, Umeå, Sweden), where log transformation, Pareto scaling and centring were applied. Two-component Principal Component Analyses (PCA) provided an overview of the data and checking the quality of the analysis. Results were then analysed by Partial Least Square Discriminant Analysis (PLS-DA) (centred, UV-scaled). Each PLS-DA model was further validated thanks to permutation tests (*n* = 100 permutations) and CV-ANOVA. For better interpretability, Orthogonal Projection to Latent Structure Discriminant Analyses (OPLS-DA) were also performed.

Univariate analysis was performed on all datasets using a Wilcoxon test in R studio and *p*-values were calculated using the *coin* package (R studio).

## 3. Results

In order to investigate changes in the lipidome profiles and the complementarity of different MS fingerprinting strategies, a set of samples from which the lipid profiles were expected to be disrupted was chosen as a proof of concept [39]. Below are described and compared the results obtained from three methods: Non-targeted RP UHPLC-HRMS and two targeted approaches, namely Lipidyzer™ and a platform focused on TG regioisomers.

### 3.1. Non-Targeted RPLC-HRMS

In the frame of a global lipidomics study, a common method is the non-targeted fingerprinting using LC-HRMS, as it allows studying a large set of lipid species without any a priori hypothesis [40], i.e., theoretically all lipids accessible to the analysis technique. In the present case, the objective was not to develop a new analytical approach but rather evaluate the contribution of an already established workflow [12] in the frame of a multi-platform study.

**Figure 1:**
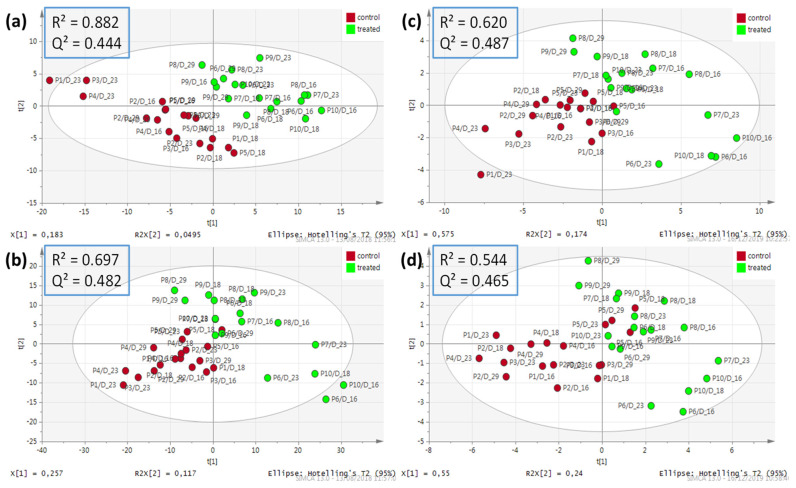
PLS-DA score plots after removing QC, D0, D3, D9 samples from the cleaned ESI− and ESI+ datasets acquired with RP UHPLC-HRMS. Datasets containing 1612 (ESI−) (**a**) and 2914 (ESI+) (**b**) features, *n* = 36. Reduced datasets containing 94 (ESI−) (**c**) and 46 (ESI+) (**d**) features, *n* = 36. Log 10 transformation, Pareto scaling and centering were applied.

After acquisition and verification of the fingerprint quality (see details in Supplementary Materials), 1612 and 2914 features were selected in the ESI− and ESI+ datasets, respectively. A PCA allowed highlighting clustering of the QC samples, thus demonstrating the reproducibility of the analysis (see Figure S1). Furthermore, in PCAs score plots, samples from D3 and D9 did not show major differences between groups, probably because of the slow response of the lipidome to such growth-promoting treatment as previously observed [39]. Consequently, these early collection points were removed, and the PCAs generated on the resulting ESI+ and ESI− datasets (D16, D18, D23, D29 samples) exhibited separation trends between groups (see Figure S2). PLS-DA were then performed and a discrimination between groups was observed (Figure 1, left panel) with the following performance: R^2^ = 0.882 and Q^2^ = 0.444 for ESI−; R^2^ = 0.697 and Q^2^ = 0.482 for ESI+. The models were further assessed with CV-ANOVA (*p*-value = 9.5 × 10^−4^ for ESI− and *p*-value = 3.1 × 10^−4^ for ESI+) indicating significant statistical models [41]. For both models, high R^2^ values demonstrated high descriptive ability, while Q^2^ values (<0.5) pointed out limited predictability, as confirmed by permutation tests (Figure S3). This was attributed to the high number of features—generating noise—while better predictive models were expected through refined selection of features. Such selection would also answer our needs in terms of classification model practical implementation. Consequently, the features of interest were determined using a strategy successfully applied in previous works based on OPLS-DA outcomes [42], here through assessment of variable importance for projection of the predictive component (VIPpred) [43], using Workflow4metabolomics 3.3 [44,45,46]. VIPpred was specifically chosen as it is purely associated with the consequences of the treatment, as opposed to the orthogonal component, associated with the experimental variability and time-related evolution of the individuals. In order to select only robust and discriminating features between the groups studied, the threshold applied to their selection (VIP pred >1.8) was deliberately chosen to be more stringent than the classically reported value of VIPpred >1.5. The consequence of such a choice was the reduction of the number of features thus selected (46 from the ESI+ datasets/94 from the ESI− vs. 374 from ESI+ and 203 from ESI−, respectively), but to the benefit of the quality of these potential biomarkers. All of these features exhibited higher signal intensity in the samples from treated animals. From these features, new PLS-DA models were built (Figure 1, right panel), showing a strong discrimination between groups, with, the following performance for the reduced ESI+ model: R^2^ = 0.544; Q^2^ = 0.465, CV-ANOVA *p*-value = 5.3 × 10^−4^; and reduced ESI− model: R^2^ = 0.620; Q^2^ = 0.487, CV-ANOVA *p*-value = 3.3 × 10^−4^. The quality of the reduced models was also confirmed by permutation tests (Figure S4).

The relevance of the selected features was confirmed by “day-by-day” Wilcoxon tests. Thanks to additional data from dependent MS/MS—Top 15 (Full MS/dd-MS^2^-Top 15) experiments performed on QC samples and four typical samples (P4 (control) and P8 (treated) at D18 and D23), a few of them could be putatively using the LipidSearch tool. Annotations and statistical results are detailed in Table 2. Detailed results from LipidSearch for these features can be found in Supplementary Materials (Table S1). From the reduced ESI− dataset, 3 PC, 8 PE and 1 phosphatidylserine (PS) could be annotated whereas 1 PC, 2 PE and 9 TG were annotated from the reduced ESI+ dataset, including 1 PE, which was annotated in both ionisation modes (PE(17:0_20:4)). From these preliminary results, it can be noticed that the discrimination between samples from control and treated animals mainly relies on phospholipids and TG, which was consistent with recent literature [13]. PC appears to be mostly discriminant (*p*-value ≤ 0.05) at D16, D18 and D29, PE at D16 and D23 and TG at D23. The annotated phosphatidylserine (PS(18:2_21:0)) was found to be discriminant at all kinetic points between D16 and D29. However, three annotated TG (TG(16:0_17:0_18:1) and the two adducts of TG(18:0_17:0_18:1)) did not exhibit *p*-values ≤ 0.05 and thus could be regarded as modestly involved in the discrimination between groups. 

### 3.2. Lipidyzer™ Platform

In order to provide additional insight into lipids involved in the sample group separation observed above, an alternative MS lipidomics approach was applied. Lipidyzer™ is a commercial lipid quantification tool based on shotgun lipidomics and benefiting from ion mobility, coming with its own workflow and dedicated framework. As it is based on targeted analysis and differs in the separation mode, complementary results from the non-targeted method presented above are expected. Lipidyzer™ was originally designed for human blood serum and plasma studies, but its applicability may be tested for other species. However, since it was used here for porcine serum samples, the associated results cannot be considered as absolute concentrations. Because of the lack of validation for pig samples, the Lipidyzer results detailed in this work were, therefore, considered as “estimated” concentration. Here, this experiment required a limited number of samples, hence samples collected at D3 (as a basis for comparison), D18 and D23 were characterised with Lipidyzer™, as a result of RPLC-HRMS outcomes described above. From the analysed samples, 795 lipid species were actually measured (i.e., above limit of quantification in at least one sample), namely: 26 Cholesterol Esters (CE), 10 Ceramides (CER), 7 Dihydroceramides (DCER), 11 Hexosyl ceramides (HCER), 10 Lactosyl ceramides (LCER), 54 Diacylglycerols (DAG), 26 Free Fatty Acids (FFA), 18 Lysophosphatidylcholine (LPC), 89 PC, 13 Lysophosphatidylethanolamines (LPE), 107 PE, 12 SM and 490 TG. Univariate statistical tests (Wilcoxon, day by day) were performed, showing significant shifts upon RAC treatment for 22 CE, 1 CER, 11 DAG, 1 DCER, 1 FFA, 3 HCER, 3 LCER, 1 LPE, 26 PC, 12 PE, 5 SM and 152 TG (see Table 3). Details about these species can be found in Table S2.

**Figure 2:**
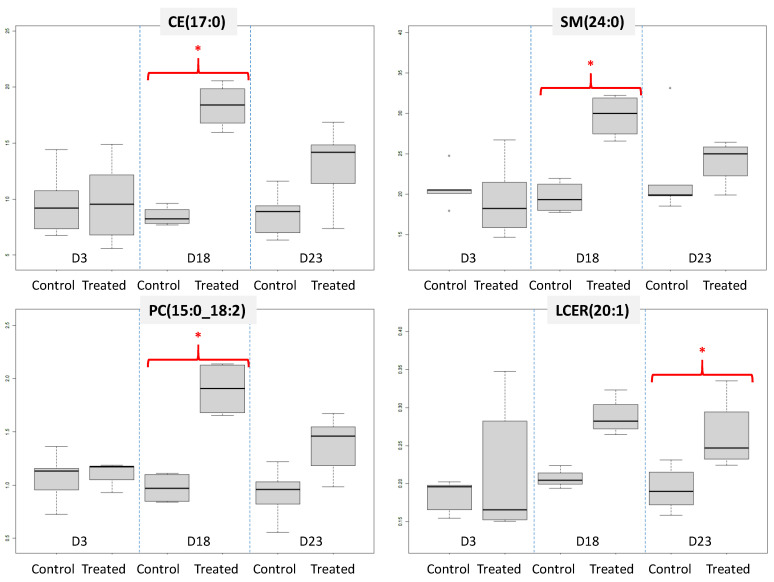
Comparison of estimated concentration (nmol·g^−1^) from four lipid species analysed with Lipidyzer™ between the two animal groups of interest, and for different serum collection points. Here, the quantification cannot be considered as accurate (hence “estimated”) since it is has not been validated on pig serum, as opposed to human. *: *p*-value ≤ 0.05.

When looking at the differences of concentration between samples from control and treated animals at D3 for all measured lipids, only 1 HCER (HCER(24:1)) and 1 PE (PE(O-18:0_18:1)) were shown as significant (*p*-value ≤ 0.05), while 1 PC (PC(16:0_18:0)) and 1 TG (TG42:1-FA14:0) were marginally significantly affected (*p*-value ≤ 0.06). This correlates non-targeted results, where no significant patterns could be observed so early in the experiment. Interestingly, CE, CER, DCER, HCER, LCER, LPE and SM species appeared as significant in the discrimination almost exclusively at D18, while DAG exhibited a significant shift in lipid profiles mainly at D23. The significant shift of species from other classes was distributed evenly between D18 and D23. All the lipids were observed to be more concentrated in the serum of treated animals, except for 1 HCER measured in lower concentration in the serum of treated animals at D3 (HCER(24:1)). Globally, when the number of significant lipid species in either D3, D18, or D23 samples was proportionated to the number of analysed species per class, the most altered classes were CE, (85% of analysed species deemed as significant), SM, (42%), TAG (31%), LCER (30%), PC (29%) and HCER (27%). Examples of boxplots illustrating differences in concentration levels between samples from control and treated groups for four particular species are presented in Figure 2.

### 3.3. TG Platform

The characterisation of the different TG isomers is an issue that was not completely addressed by Lipidyzer™, which justified resorting to a dedicated TG platform, originally developed for the annotation and semi-quantification of TG isomers in vegetable oils [35].

Through modelling of the fragmentation patterns in TG containing common fatty acids, using multivariate constrained regression, this TG platform was able to determine their regioisomeric composition. This analytical method is semi-quantitative and aimed at highlighting TG patterns, together with their fatty acid composition. Relative proportions for each regioisomer (TG(rac-A/B/C); A, B and C corresponding to the constituting fatty acyl chains) can also be determined. The analysis was performed on a limited number of relevant samples: D3 as a reference and samples from D16 to D29, corresponding to time points for which most important TG shifts had been observed using both previous platforms.

From univariate statistical tests (Table 4), five TG (TG(52:5), two TG(54:6), TG(54:5) and TG(54:7)) were detected as significant (Wilcoxon test) in the context of the study for the discrimination between control and treated sample groups at D23, with higher concentrations upon RAC treatment. Two of them, namely TG(54:6) at the retention time (Rt) 555.9 s and TG(54:7), were also found as significant at D16, but with a limit *p*-value (0.05) and slightly lower concentrations in treated individuals. For detected TGs, the proportions of the corresponding regioisomers can also be estimated. For instance, the significant variable TG(54:7), detected at Rt 476.03 s was mainly constituted by TG(rac-18:2/18:2/18:3) (around 60%) but also TG(rac-18:2/18:3/18:2) (around 40%).

## 4. Discussion

### 4.1. Assessment of the Complementarity between Platforms

Three platforms differently addressing the lipidome were involved in the characterisation of a set of serum samples in which specific lipid patterns are expected to be observed. The results have been carefully compared for assessing their respective contributions and complementarity in lipidomics in general and for the proposed application. As a preliminary step, reproducibility was compared between the platforms, which were assessed by CV(QC)% on common lipid targets (*n* = 30), resulting in median values below 8%, which were considered to fit our requirements.

Whatever the platform used, the disruption of various lipid classes could be highlighted in pig serum after several weeks of RAC treatment, as illustrated in Table 5. The same trends could be observed with the three tools, as higher lipid levels were observed in the serum of treated individuals, e.g., for TG (non-targeted, Lipidyzer™ and TG platforms) but also for PC and PE (non-targeted and Lipidyzer™ platforms). A graphical illustration of these shared trends can be found in Figure S5.

To check the consistency between these results, the annotated lipids highlighted by the reduced models in the non-targeted analysis were searched in Lipidyzer™ outcomes. Most of them could easily be retrieved and were also found to be significant (*p*-value <0.05) with the same variations towards RAC treatment, highlighting good consistency, in particular for PC(15:0_18:1), PC(17:0_18:1), PC(18:1_14:0) PE(16:0_20:4); PE(16:0_18:2) and PE(16:0p_20:4). The collection dates when these lipids were found to be significant were generally in accordance, although minor differences were observed. For instance, PC(18:1_14:0) was only highlighted at D18 with the non-targeted analysis, whereas it was also found to be marginally significant at D23 (*p*-value = 0.056), using Lipidyzer™ (as “PC(14:0_18:1)”). Still, some lipids that were highlighted with the non-targeted approach were not observed as significant with Lipidyzer™, usually due to a corresponding signal below the limit of quantification with the latter, as observed for PE(17:0_20:4). In other cases, the reason for this difference was less clear; e.g., PE(16:0_18:1) and PE(16:0p_22:4), which were retained from ESI− non-targeted results were not found as significant with Lipidyzer™. This could be explained by different measurement biases or by erroneous annotation, even if no obvious inconsistency was observed. Conversely, significant Lipidyzer™ features were curated in the non-targeted datasets. Even though some lipid classes from which the lipid species were deemed as significant by Lipidyzer™ (*p*-value ≤ 0.05) were annotated in the non-targeted dataset, some did not belong to the set of features selected for the reduced model. Indeed, CE and DAG were detected and annotated in the ESI+ dataset, LPE and FFA were observed in the ESI− datasets, whereas CER, DCER and SM were characterised in both.

An important matter to consider when comparing the results between platforms is their relative capability for lipid annotation, which as a consequence, directly influences the biological interpretation.

With the non-targeted strategy proposed, the annotation is only putative (level 2 or 3 of identification), and a small portion (<20% for both datasets) of the original features could be annotated, thus demonstrating the challenge of this step. Using targeted approaches, such an issue is less likely to happen as their workflows were optimised to target specific lipids of interest. Implementing Lipidyzer™ and the TG platforms thus enabled confident lipid assignment.

While comparing the platform’s outcomes and lipid annotation, a particular case is the one of TG, where the assignment of the fatty acyl chains (*sn*-1(3) versus *sn*-2) is recognised as a serious analytical challenge, leading to multiple dedicated research studies [47,48,49].

- From the non-targeted method, TGs were annotated from their three FA chains (e.g., “TG(16:0_17:0_18:1)”), based on the annotation results from LipidSearch after data-dependent MS/MS. Although allowing confident assignment, the results of such an approach may in some particular cases be considered with caution as illustrated hereafter. Among the selected features, for instance, some lipids (M926T1080 and M921T1080; highlighted in light grey as well as M898T1065 and M893T1066 highlighted in dark grey in Table 2) were annotated as adducts of the same TG. These features were initially not discarded during the data processing step because of an inconsistency between the adduct annotation between the CAMERA package and LipidSearch. In addition, two other features (M919T1066 and M924T1066; highlighted in blue in Table 2) were annotated as two different TG when they could potentially be two adducts of the same lipid as they are isomers of TG(55:2).
- In Lipidyzer™, TG results were expressed with the shorthand annotation nomenclature (total number of carbons and unsaturations among the three FA chains and the precision on one of them), such as ”TG51:1-FA16:0”. While technically correct, this leads to an overestimation of the TG, as previously highlighted in the literature [31]. Moreover, several Lipidyzer™ candidates (e.g., TG51:1-FA18:1 and TG51:1-FA16:0) can correspond to a single TG feature in RP LC-HRMS (e.g., TG(16:0_17:0_18:1)), and vice-versa, thus complicating result comparison.
- Because of previous issues in TG assignment, a dedicated platform for the determination of TG regioisomeric composition was used [35]. It is interesting to note that the TGs highlighted with the dedicated tool were not those annotated in non-targeted data. Moreover, after conversion to the corresponding shorthand annotation to allow such a comparison, none of them was deemed as significant with Lipidyzer™, which could be due to the overestimation of TG with the latter. Conversely, none of the discriminant TG highlighted within the RPLC-HRMS results were monitored with the TG platform since it is designed for the analysis of even FA chains TG only. It is interesting to note that this specific platform allowed obtaining confident results on TG and the position (*sn*-1(3) versus *sn*-2) of their constituting FA chains. Thus, it yielded finer results than the combined use of non-targeted and Lipidyzer platforms—an approach that was already explored by Contrepois et al. [31].

Between all evaluated platforms, Lipidyzer™ offered the most detailed analysis for a large number of lipids, providing a large amount of biologically interpretable data. Yet, interpretation issues were observed when considering the TG because of the overestimated occurrence of this class, whereas the TG platform could bring information on the regioisomers of interest without doubt. However, the latter was designed for this class only, and the number of followed species and regioisomers is limited.

Nonetheless, targeted platforms focus on a limited number of lipids, originally selected for a particular application, i.e., the human serum/plasma studies for Lipidyzer™ and vegetable oils for the TG platform. Hence, the relevance of the monitored compounds is not guaranteed when applied to a different research question, and species of interest are also likely to be overlooked, as opposed to the non-targeted strategy. For instance, applying the latter enabled highlighting PS(18:2_21:0) in ESI− as well as PC(15:0_16:0) and PC(16:0_19:0) in ESI+ as relevant upon RAC treatment.

Regarding practical considerations, Lipidyzer can be performed in an easy manner, thanks to the entirely software-guided workflow, from instrument calibration to processing. Comparatively, the non-targeted platform requires more expertise, in particular for data processing, even though tools are available to make this step more accessible, such as Workflow4Metabolomics (W4M) [44,45]. Since it is still recent, the TG platform still requires a high level of expertise for using the dedicated in-house R algorithm.

Among the three platforms, Lipidyzer™ can be considered as the quickest since the analysis (two 15-min injections, comparable with the 30-min of the non-targeted method and 18-min of TG platform) is compensated by the assisted data processing, allowing a direct interpretation of the results. Nevertheless, since it entails the purchase of dedicated instrument/software/kits, Lipidyzer™ implies a substantial financial investment, whereas the other two can be adapted to various instrument types, although buying pure standards is still required for the confirmation of lipid assignment or the calibration of TG regioisomers.

The investigation of the serum lipidome disruptions upon RAC administration to pigs showed the added value of the three tools. Rather than heaving up one particular platform above the others, these results clearly demonstrate how comprehensive lipidome characterisation is a challenging task, requiring several tools for both enhanced lipidic coverage and increased confidence in the observations.

### 4.2. Biological Interpretation

The results enabled further investigating ractopamine effects on pig blood lipids profile. Although a full biological interpretation of the metabolic pathways was out of the scope of this work, our observations are discussed below in light of the current knowledge regarding the impact of RAC on metabolism.

RAC is a synthetic drug belonging to the β-agonist family, widely used as a growth promoter in several countries, as it has been shown to improve growth performance such as average daily gain [50] in pigs. However, as such, it is banned in the European Union [1,51], and robust screening methods are required to detect any potential abuse. In such context, metabolomics has been successfully applied for screening β-agonists treatment in bovine, thus highlighting the signature of administration, enabling the construction of new robust models based on these biomarkers [42]. That is why RAC effects have been similarly studied in porcine, using non-targeted tissue screening [13] and serum metabolomics [32]. As the lipids are known to be disrupted by the use of this compound [52,53,54], the lipidome appears as a promising compartment for inspecting the effects of RAC, prompting their study by NMR lipidomics [39] and the currently presented work. The mechanism of action of RAC as a growth promoter is relatively well-known; it stimulates β_2_-adrenergic receptors, linked with the relaxation of smooth muscles. They enhance the synthesis and decrease the degradation of proteins [55]. A reduction of adipose tissues as an effect of RAC treatment is commonly reported through two pathways: reduction of lipogenesis and/or increase in lipolysis, as reported by Ferreira et al. [54]. This review concluded on a predominance of the former, as a treatment generally does not induce an increase of serum non-esterified fatty acids (NEFA), which is characteristic of the latter.

As observed above, the blood serum levels of various lipid classes appear to be affected by the RAC treatment, starting on the third week of the experiment. The disrupted phospholipid profiles observed in the present study are in accordance with the NMR study [39] and previous observations on muscle where modified diacylglycerophosphoethanolamine and phosphatidylinositol profiles have been associated with RAC administration to pigs [13]. Among the highlighted classes, the disruption of SM is in accordance with reported observations in tissue, associating changes in sphingomyelin profiles with RAC administration to pigs [13]. For all involved lipid classes, a delay in the action of RAC can be observed. Further, a limited effect is observed at D29, although the animals were still exposed to the drug. Such observation could be hypothesised to be linked with a de-sensitisation regarding the RAC treatment, which occurs from 21 to 28 days, according to Ferreira et al. [54]. Interestingly, some odd-numbered fatty acids such as C17-cholesteryl esters and C-15 containing phosphatidyl choline were highlighted as modified upon ractopamine treatment, which is quite unexpected as almost all natural occurring fatty acids are even-numbered, although some odd-numbered fatty acids also exist. The metabolism of odd-numbered fatty acids is, however, specific in that they are reported not to be favorable substrates for beta-oxidation-related enzymes, thus leading to accumulation in the tissues [56].

These observations could form the basis for a better understanding of the mechanism underlying β-agonist treatment on lipid metabolism. Here, no particular effect of RAC could be observed on the free FA profiles (covered by Lipidyzer™). Hence, even if a deeper biological interpretation is necessary before drawing definitive conclusions, this seems in accordance with Ferreira’s review [54], suggesting inhibition of lipogenesis as a preferential mechanism of the effect of RAC, rather than an increase in lipolysis, which would have conducted to higher free FAs levels in the blood.

## 5. Conclusions

This work describes the combination of three different fingerprinting approaches in order to join their forces for one single study dedicated to food safety. Serum samples from an animal experiment involving a repartitioning agent of interest were characterised. This combination allowed a fine characterisation of the lipid profiles, showing particular lipid classes and species disruptions in pig blood serum following RAC treatment. Specific benefits could be highlighted from the three described platforms in terms of lipidome coverage, level of characterisation or applicability. Although these platforms enabled reaching complementary information, further work should be conducted to validate the proposed workflows.

For optimising lipidome characterisation, the next refinements of the strategy will be directed towards the improvement of lipid annotation from non-targeted RP UPLC-HRMS. Many tools have been reported in the recent literature such as LOBSTAHS [57] or LipidMatch [58], and their evaluation/implementation would ensure higher confidence in results and facilitated link with other platforms. The selection of relevant features could also be improved, through the use of sparse methods [59,60] or the recent *biosigner* algorithm [61], precisely aiming at building reduced models. Moreover, the TG platform could be extended in order to include more lipid species, thus requiring further developments in order to increase its suitability to a wider range of lipidomics applications. Improvements could also be made for the development of a more user-friendly data processing interface, which would make this platform accessible to less-experienced analysts and accelerate the time dedicated for such data handling.

Regarding the study of the effects of RAC on pig’s lipidome, further work is still necessary to fully understand the biological implications underlined by the presented results. Additional animal experiments could also be performed involving, for instance, different dosages or individuals with different characteristics, for confirming these outcomes and validate candidate biomarkers. From a public health perspective, it is expected that the outcomes of the present study may serve risk analysis, either at the risk assessment level while proposing new insight on the mode of action and associated effects or at the risk management steps, as the basis for an alternative screening method based on lipid biomarkers.

## Figures and Tables

**Table 1:** Characteristics of the three used platforms and associated experimental details.

Platform	Non-Targeted RPLC-HRMS [12]	Targeted Lipidyzer™ [33,34]	Targeted TG Platform [35]
Extraction type	Bligh and Dyer—like [12]	Two solvent addition/organic phase transfer cycles	Bligh and Dyer—[12]
Samples	D3, D9, D16, D18, D23 and D29 QC	D3, D18 and D23 QC Lipidyzer-specific QC and QC spike samples	D3, D16, D18, D23 and D29 QC
Serum volume	30 µL	30 µL	10 µL, completed with 20 µL H_2_O
Solvents	Methanol (MeOH), Chloroform (CHCl_3_), Water (H_2_O)	MeOH, dichloromethane (DCM), H_2_O	MeOH, CHCl_3_, H_2_O
Centrifugation	Yes	Yes, two times	Yes
Internal standards	*n* = 7 In CHCl_3_, 0.5 mg·L^−1^	Lipidyzer™ standard kit, *n* = 54 30 µL added at beginning (See Supplementary Materials)	*n* = 3 In CHCl_3_, 0.132 µmol·L^−1^
Transfer	200 µL organic phase	Multiple organic phases	200 µL organic phase
Evaporation	Yes	Yes	Yes
Reconstitution solvent	Acetonitrile(AcN):Isopropanol(IPA):H_2_O (65:30:5, v:v:v)	DCM:MeOH (50:50, v:v), 10 mM Ammonium Acetate	AcN:IPA (50:50, v:v)
Reconstitution volume	200 µL	300 µL	200 µL
Analysis Technique	LC-HRMS (full-scan + data dependent MS/MS)	DMS-MS/MS (direct introduction)	LC-MS/MS
Quantification	No	Yes	No
Targeted	No	Yes	Yes
Analytical system	LC: Thermo UltiMate^®^ 3000 MS: Thermo Q-Exactive	Sciex QTRAP 5500, with SelexION differential mobility spectrometry (DMS)	LC: Waters Acquity UPLC MS: Waters Acquity-Synapt G2S Q-TOF
Column	Waters CSH C18 (100 × 2.1 mm i.d., 1.7 µm particle size)	None (direct introduction)	Waters BEH C18 (150 × 2.1 mm i.d. 1.7 µm particle size)
Mobile phase	A: ACN:H_2_O (60:40, v:v) B: IPA:ACN:H_2_O (88:10:2, v:v:v) Both: 10 mM ammonium acetate + 0.1% acetic acid	DCM:MeOH (50:50, v:v) 10 mM Ammonium Acetate	A: MeOH B: MeOH/IPA (50:50, v:v) Both: 2 mM ammonium acetate + 6 mM acetic acid
Ionisation	Polarity switching mode ESI− and ESI+	Polarity switching mode ESI− and ESI+	ESI+
Data processing	MSConvert [36] XCMS [37], CAMERA Batch drift correction [38] Annotation: Lipidsearch (Thermo Fisher Scientific) after additional data dependent MS/MS—Top 15 (Full MS/dd-MS^2^-Top 15) acquisitions	Automated Lipidyzer™ framework	MassWolf XCMS [37] In-house R algorithm
Number of features/lipids in analysed samples	ESI−: 1612 features ESI+: 2914 features	873 lipids *	50 TG **
Quality Assurance/Quality Control	Randomisation, QC (pooled samples), Internal standards, Extraction blanks	Randomisation, QC (pooled samples), Control plasma, Spiked samples, Internal standards, Extraction blanks	Cross checking of platform performance [35], calibration, QC (pooled samples), extraction blanks

* 383 individual species + 490 TG, including redundancies (see details in appropriate section) ** 143 regioisomers in total when considering proportion estimates (see details in appropriate section).

**Table 2:** Putatively annotated features of interest extracted from the reduced the LC-HRMS datasets, with associated VIPpred values from the OPLS-DA used for variable selection and *p*-values from a Wilcoxon test. **: *p*-value < 0.01; *: *p*-value ≤ 0.05. ^†^: VIPpred values from the OPLS-DA model based on the 1612 (ESI−) and 2914 features (ESI+) after removal of QC, D0, D3 and D9.

Variable ID	VIPpred ^†^	Annotation (LipidSearch)	MS^2^ Validation (LipidSearch)	*p*-Value D16	*p*-Value D18	*p*-Value D23	*p*-Value D29
ESI−							
M791T491	1.81	[PC(18:1_14:0) + CH_3_COO]^−^	✓	0.117	* 0.027	0.117	* 0.034
M805T538	1.98	[PC(15:0_18:1) + CH_3_COO]^−^	✓	** 0.009	* 0.014	* 0.028	* 0.034
M833T633	1.84	[PC(17:0_18:1) + CH_3_COO]^−^	✓	* 0.028	* 0.014	0.076	* 0.034
M715T534	1.92	[PE(16:0_18:2)-H]^−^	✓	0.117	0.221	** 0.009	0.480
M717T611	2.18	[PE(16:0_18:1)-H]^−^	✓	* 0.028	0.806	** 0.009	0.480
M739T518	1.97	[PE(16:0_20:4)-H]^−^	✓	* 0.047	0.086	* 0.016	0.480
M745T705	2.05	[PE(18:0_18:1)-H]^−^	✓	0.076	0.142	* 0.047	0.289
M753T566	2.02	[PE(17:0_20:4)-H]^−^	✓	* 0.028	* 0.014	0.076	0.480
M765T524	2.17	[PE(18:1_20:4)-H]^−^	✓	0.076	0.142	* 0.016	0.157
M723T563	1.90	[PE(16:0p_20:4)-H]^−^	✓	** 0.009	0.221	** 0.009	0.077
M751T659	1.84	[PE(16:0p_22:4)-H]^−^	✓	** 0.009	0.327	* 0.028	0.157
M829T472	1.80	[PS(18:2_21:0)-H]^−^	✓	* 0.016	* 0.050	* 0.028	* 0.034
ESI+							
M777T719	1.95	[PC(16:0_19:0) + H]^+^	X	* 0.047	* 0.027	0.175	0.289
M755T566	1.81	[PE(17:0_20:4) + H]^+^	✓	** 0.009	* 0.014	* 0.047	0.077
M759T836	1.84	[PE(20:0p_18:1) + H]^+^	✓	* 0.028	* 0.050	* 0.047	0.077
M865T1051	1.87	[TG(16:0_17:0_18:1) + NH_4_]^+^	✓	0.117	0.086	0.076	0.157
M879T1059	1.92	[TG(18:0_16:0_18:1) + NH4]^+^	✓	0.076	0.142	* 0.028	0.157
M891T1051	1.84	[TG(17:0_18:1_18:1) + NH_4_]^+^	✓	0.117	0.086	* 0.047	0.157
M893T1066	1.91	[TG(18:0_17:0_18:1) + NH_4_]^+^	✓	0.076	0.086	0.076	0.289
M898T1065	1.84	[TG(18:0_17:0_18:1) + Na]^+^	✓	0.117	0.086	0.076	0.157
M921T1080	2.35	[TG(18:0_18:1_19:0) + NH_4_]^+^	✓	0.117	0.086	* 0.047	0.157
M926T1080	1.99	[TG(18:0_18:1_19:0) + Na]^+^	✓	* 0.047	0.086	0.076	0.157
M919T1066	1.88	[TG(19:1_18:0_18:1) + NH_4_]^+^	✓	* 0.047	0.142	* 0.016	0.077
M924T1066	1.84	[TG(19:0_18:1_18:1) + Na]^+^	✓	0.076	0.142	* 0.047	0.157

**Table 3:** Lipid class analysis results from Lipidyzer™, with associated *p*-values from a Wilcoxon test. **: *p*-value ≤ 0.01; *: *p*-value ≤ 0.05.

Lipid Class	*p*-Value D3	*p*-Value D18	*p*-Value D23
CE	0.55	*0.03	0.10
CER	0.22	0.11	0.31
DAG	0.42	0.20	** 0.01
DCER	0.42	1.00	0.22
FFA	1.00	0.20	0.42
HCER	0.15	* 0.03	0.69
LCER	0.69	* 0.03	** 0.01
LPC	0.06	0.34	0.84
LPE	0.15	0.11	0.55
PC	0.69	0.06	0.06
PE	0.84	* 0.03	** 0.01
SM	0.22	* 0.03	1.00
TG	0.55	0.20	0.06

**Table 4:** Results from the TG platform, with associated *p*-values from a Wilcoxon test. *: *p*-value ≤ 0.05. For each TG signal, the corresponding regioisomers and associated estimated proportions are detailed. The main regioisomers are in bold.

TG_Rt	Corresponding Regioisomers with Estimated Proportions	*p*-Values				
		D3	D16	D18	D23	D29
TG(52:5)_553.44s	TG(rac-18:3/16:0/18:2)~15% **TG(rac-16:0/18:2/18:3)**~**50%** TG(rac-16:0/18:3/18:2)~35%	0.44	0.77	0.64	* 0.03	0.06
TG(54:6)_555.9s	**TG(18:2/18:2/18:2)**	0.17	* 0.05	0.39	* 0.03	0.72
TG(54:6)_566.5s	**TG(rac-18:3/18:1/18:2)**~**60%** TG(rac-18:1/18:2/18:3)~10% TG(rac-18:1/18:3/18:2)~30%	0.17	0.18	0.25	*0.03	1.00
TG(54:5)_685.8s	**TG(rac-18:3/18:0/18:2)**~**60%** TG(rac-18:0/18:2/18:3)~20% TG(rac-18:0/18:3/18:2)~20%	1.00	0.65	0.15	* 0.05	0.51
TG(54:7)_476.03s	**TG(rac-18:2/18:2/18:3)**~**60%** TG(rac-18:2/18:3/18:2)~40%	0.65	* 0.05	0.64	* 0.03	1.00

**Table 5:** Comparison of the results from various MS platform. The analysed lipid classes are mentioned with the level of significance, determined from a univariate Wilcoxon test.

	Non-Targeted RP LC-HRMS		Lipidyzer™		TG Platform	
Class of the Relevant Lipids	Analysed and Annotated?	Variation (If Significant)	Analysed and Annotated?	Variation (If Significant)	Analysed and Annotated?	Variation (If Significant)
CE	Yes ^†^		Yes	↗D18 * ↗ D23 *	No	-
CER	Yes ^†^		Yes	↗ D18 *	No	-
DAG	Yes ^†^		Yes	↗ D18 * ↗ D23 *	No	-
DCER	Yes ^†^		Yes	↗ D18 *	No	-
FFA	Yes ^†^		Yes	↗ D18 * ↗ D23 *	No	-
HCER	Yes ^†^		Yes	↘ D3 * ↗ D18 *	No	-
LCER	No		Yes	↗D18 * ↗ D23 *	No	-
LPC	Yes ^†^		Yes	-	No	-
LPE	Yes ^†^		Yes	↗ D18 *	No	-
PC	Yes	↗ D16 *, ↗ D18 *, ↗ D23 *, ↗ D29 *	Yes	↗ D18 * ↗ D23 *	No	-
PE	Yes	↗ D16 *, ↗ D18 *, ↗ D23 *	Yes	↗ D3 * ↗ D18 * ↗ D23 *	No	-
PS	Yes	↗ D16 *, ↗ D18 *, ↗ D23 *, ↗ D29 *	No	-	No	-
SM	Yes ^†^		Yes	↗ D18 *	No	-
TG	Yes	↗ D16 *, ↗ D23 *	Yes	↗ D18 * ↗ D23 *	Yes	↘ D16 *, ↗ D23 *

Level of significance after Wilcoxon test is indicated with asterisks: *: *p*-value ≤ 0.05. ^†^: Lipid class analysed and annotated by non-targeted RP UPLC-HRMS but not observed in the set of selected features from OPLS-DA (VIPpred > 1.8). ↘: More concentrated in control samples. ↗: More concentrated in samples from treated animals. In bold: Days where main disruptions are observed.

## Data Availability

Not applicable.

## Supplementary Materials

The following are available online at https://www.mdpi.com/article/10.3390/foods10061218/s1, Figure S1: PCA Score plot (with QC) from the non-targeted RP LC-HRMS datasets, Figure S2: PCA Score plot (Without QC, Day-3 and Day-9 samples) from the non-targeted RP LC-HRMS datasets, Figure S3: Permutation tests (*n* = 100 permutations) associated with the PLS-DA models (Without QC, Day-3 and Day-9 samples) from the non-targeted RP LC-HRMS datasets, Figure S4: Permutation tests (*n* = 100 permutations) associated with the reduced PLS-DA models (Without QC, Day-3 and Day-9 samples) from the non-targeted RP LC-HRMS datasets, Figure S5: Intensity trend comparison of two lipid species analysed with RPLC-HRMS (ESI−) and Lipidyzer in control and treated samples from D23, Figure S6: m/z measurement error (ppm) on the internal standards signals from QC samples injections, Table S1: Results from the LipidSearch annotation after MS^2^ analysis for the features of interest, Table S2: Statistical results from the Lipidyzer platform.

## References

[1] Council Directive 88/146/EEC Council Directive 88/146/EEC Prohibiting the Use Livestock Farming of Certain Substances Having a Hormonal Action. http://data.europa.eu/eli/dir/1988/146/oj.

[2] European Parliament and Council (2017). Regulation (EU) 2017/625 on Official Controls and Other Official Activities Performed to Ensure the Application of Food and Feed Law, Rules on Animal Health and Welfare, Plant Health and Plant Protection Products, Amending Regulations (EC) No 999/2001, (EC) No 396/2005, (EC) No 1069/2009, (EC) No 1107/2009, (EU) No 1151/2012, (EU) No 652/2014, (EU) 2016/429 and (EU) 2016/2031 of the European Parliament and of the Council, Council Regulations (EC) No 1/2005 and (EC) No 1099/2009 and Council Directives 98/58/EC, 1999/74/EC, 2007/43/EC, 2008/119/EC and 2008/120/EC, and repealing Regulations (EC) No 854/2004 and (EC) No 882/2004 of the European Parliament and of the Council, Council Directives 89/608/EEC, 89/662/EEC, 90/425/EEC, 91/496/EEC, 96/23/EC, 96/93/EC and 97/78/EC and Council Decision 92/438/EEC (Official Controls Regulation). OJ L 95, 7.4. http://data.europa.eu/eli/reg/2017/625/oj.

[3] Pinel G., Weigel S., Antignac J.-P., Mooney M., Elliott C., Nielen M., Le Bizec B. (2010). Targeted and untargeted profiling of biological fluids to screen for anabolic practices in cattle. TrAC Trends Anal. Chem..

[4] Gallart-Ayala H., Chéreau S., Dervilly-Pinel G., Le Bizec B. (2015). Potential of mass spectrometry metabolomics for chemical food safety. Bioanalysis.

[5] Courant F., Pinel G., Bichon E., Monteau F., Antignac J.-P., Le Bizec B. (2009). Development of a metabolomic approach based on liquid chromatography-high resolution mass spectrometry to screen for clenbuterol abuse in calves. Analyst.

[6] Stella R., Dervilly-Pinel G., Bovo D., Mastrorilli E., Royer A.-L., Angeletti R., Le Bizec B., Biancotto G. (2017). Metabolomics analysis of liver reveals profile disruption in bovines upon steroid treatment. Metabolomics.

[7] Lu H., Zhang H., Zhu T., Xiao Y., Xie S., Gu H., Cui M., Luo L. (2017). Metabolic Effects of Clenbuterol and Salbutamol on Pork Meat Studied Using Internal Extractive Electrospray Ionization Mass Spectrometry. Sci. Rep..

[8] Dervilly-Pinel G., Courant F., Chéreau S., Royer A.-L., Boyard-Kieken F., Antignac J.-P., Monteau F., Le Bizec B. (2012). Metabolomics in food analysis: Application to the control of forbidden substances. Drug Test. Anal..

[9] Dervilly-Pinel G., Weigel S., Lommen A., Chereau S., Rambaud L., Essers M., Antignac J.-P., Nielen M.W., Le Bizec B. (2011). Assessment of two complementary liquid chromatography coupled to high resolution mass spectrometry metabolomics strategies for the screening of anabolic steroid treatment in calves. Anal. Chim. Acta.

[10] Jacob C.C., Dervilly-Pinel G., Biancotto G., Monteau F., Le Bizec B. (2014). Global urine fingerprinting by LC-ESI(+)-HRMS for better characterization of metabolic pathway disruption upon anabolic practices in bovine. Metabolomics.

[11] Nzoughet J.J.K., Dervilly-Pinel G., Chéreau S., Biancotto G., Monteau F., Elliott C.T., Le Bizec B. (2015). First insights into serum metabolomics of trenbolone/estradiol implanted bovines; screening model to predict hormone-treated and control animals’ status. Metabolomics.

[12] Nzoughet J.K., Gallart-Ayala H., Biancotto G., Hennig K., Dervilly-Pinel G., Le Bizec B. (2015). Hydrophilic interaction (HILIC) and reverse phase liquid chromatography (RPLC)–high resolution MS for characterizing lipids profile disruption in serum of anabolic implanted bovines. Metabolomics.

[13] Guitton Y., Dervilly-Pinel G., Jandova R., Stead S., Takats Z., Le Bizec B. (2018). Rapid evaporative ionisation mass spectrometry and chemometrics for high-throughput screening of growth promoters in meat producing animals. Food Addit. Contam. Part A.

[14] Wenk M.R. (2010). Lipidomics: New Tools and Applications. Cell.

[15] Ryan E., Reid G.E. (2016). Chemical Derivatization and Ultrahigh Resolution and Accurate Mass Spectrometry Strategies for “Shotgun” Lipidome Analysis. Accounts Chem. Res..

[16] Zhao Y.-Y., Wu S.-P., Liu S., Zhang Y., Lin R.-C. (2014). Ultra-performance liquid chromatography–mass spectrometry as a sensitive and powerful technology in lipidomic applications. Chem. Interact..

[17] Li M., Yang L., Bai Y., Liu H. (2014). Analytical Methods in Lipidomics and Their Applications. Anal. Chem..

[18] Bligh E.G., Dyer W.J. (1959). A rapid method of total lipid extraction and purification. Can. J. Biochem. Physiol..

[19] Folch J., Ascoli I., Lees M., Meath J., LeBaron F. (1951). Preparation of lipide extracts from brain tissue. J. Biol. Chem..

[20] Tumanov S., Kamphorst J.J. (2017). Recent advances in expanding the coverage of the lipidome. Curr. Opin. Biotechnol..

[21] Yang K., Han X. (2016). Lipidomics: Techniques, Applications, and Outcomes Related to Biomedical Sciences. Trends Biochem. Sci..

[22] Hu C., van der Heijden R., Wang M., van der Greef J., Hankemeier T., Xu G. (2009). Analytical strategies in lipidomics and applications in disease biomarker discovery. J. Chromatogr. B.

[23] Triebl A., Hartler J., Trötzmüller M., Köfeler H.C. (2017). Lipidomics: Prospects from a technological perspective. Biochim. Biophys. Acta (BBA) Mol. Cell Biol. Lipids.

[24] Han X., Gross R.W. (2003). Global analyses of cellular lipidomes directly from crude extracts of biological samples by ESI mass spectrometry: A bridge to lipidomics. J. Lipid Res..

[25] Schwudke D., Oegema J., Burton L., Entchev E., Hannich J.T., Ejsing C.S., Kurzchalia T., Shevchenko A. (2006). Lipid Profiling by Multiple Precursor and Neutral Loss Scanning Driven by the Data-Dependent Acquisition. Anal. Chem..

[26] Schwudke D., Schuhmann K., Herzog R., Bornstein S.R., Shevchenko A. (2011). Shotgun Lipidomics on High Resolution Mass Spectrometers. Cold Spring Harb. Perspect. Biol..

[27] Almeida R., Pauling J.K., Sokol E., Hannibal-Bach H.K., Ejsing C.S. (2014). Comprehensive Lipidome Analysis by Shotgun Lipidomics on a Hybrid Quadrupole-Orbitrap-Linear Ion Trap Mass Spectrometer. J. Am. Soc. Mass Spectrom..

[28] Hinz C., Liggi S., Griffin J.L. (2018). The potential of Ion Mobility Mass Spectrometry for high-throughput and high-resolution lipidomics. Curr. Opin. Chem. Biol..

[29] Lee H.-C., Yokomizo T. (2018). Applications of mass spectrometry-based targeted and non-targeted lipidomics. Biochem. Biophys. Res. Commun..

[30] Cajka T., Fiehn O. (2016). Toward Merging Untargeted and Targeted Methods in Mass Spectrometry-Based Metabolomics and Lipidomics. Anal. Chem..

[31] Contrepois K., Mahmoudi S., Ubhi B.K., Papsdorf K., Hornburg D., Brunet A., Snyder M. (2018). Cross-Platform Comparison of Untargeted and Targeted Lipidomics Approaches on Aging Mouse Plasma. Sci. Rep..

[32] Peng T., Royer A.-L., Guitton Y., Le Bizec B., Dervilly-Pinel G. (2017). Serum-based metabolomics characterization of pigs treated with ractopamine. Metabolomics.

[33] Lintonen T.P.I., Baker P.R.S., Suoniemi M., Ubhi B.K., Koistinen K.M., Duchoslav E., Campbell J.L., Ekroos K. (2014). Differential Mobility Spectrometry-Driven Shotgun Lipidomics. Anal. Chem..

[34] Ubhi B.K., Conner A., Duchoslav E., Evans A., Robinson R., Wang L., Baker P.R., Watkins S. (2016). A Novel Lipid Screening Platform that Provides a Complete Solution for Lipidomics Research.

[35] Balgoma D., Guitton Y., Evans J.J., Le Bizec B., Dervilly-Pinel G., Meynier A., David B., Yann G., Jason J.E., Bruno L.B. (2019). Modeling the fragmentation patterns of triacylglycerides in mass spectrometry allows the quantification of the regioisomers with a minimal number of standards. Anal. Chim. Acta.

[36] Kessner D., Chambers M., Burke R., Agus D., Mallick P. (2008). ProteoWizard: Open source software for rapid proteomics tools development. Bioinformatics.

[37] Smith C.A., Want E.J., O’Maille G., Abagyan R., Siuzdak G. (2006). XCMS: Processing Mass Spectrometry Data for Metabolite Profiling Using Nonlinear Peak Alignment, Matching, and Identification. Anal. Chem..

[38] Van Der Kloet F.M., Bobeldijk I., Verheij E.R., Jellema R. (2009). Analytical Error Reduction Using Single Point Calibration for Accurate and Precise Metabolomic Phenotyping. J. Proteome Res..

[39] Marchand J., Martineau E., Guitton Y., Le Bizec B., Dervilly-Pinel G., Giraudeau P. (2018). A multidimensional 1H NMR lipidomics workflow to address chemical food safety issues. Metabolomics.

[40] Navas-Iglesias N., Carrasco-Pancorbo A., Cuadros-Rodríguez L. (2009). From lipids analysis towards lipidomics, a new challenge for the analytical chemistry of the 21st century. Part II: Analytical lipidomics. TrAC Trends Anal. Chem..

[41] Eriksson L., Trygg J., Wold S. (2008). CV-ANOVA for significance testing of PLS and OPLS^®^ models. J. Chemom..

[42] Dervilly-Pinel G., Chereau S., Cesbron N., Monteau F., Le Bizec B. (2015). LC-HRMS based metabolomics screening model to detect various β-agonists treatments in bovines. Metabolomics.

[43] Galindo-Prieto B., Eriksson L., Trygg J. (2014). Variable influence on projection (VIP) for orthogonal projections to latent structures (OPLS). J. Chemom..

[44] Giacomoni F., Le Corguillé G., Monsoor M., Landi M., Pericard P., Pétéra M., Duperier C., Tremblay-Franco M., Martin J.-F., Jacob D. (2015). Workflow4Metabolomics: A collaborative research infrastructure for computational metabolomics. Bioinformatics.

[45] Guitton Y., Tremblay-Franco M., Le Corguillé G., Martin J.-F., Pétéra M., Roger-Mele P., Delabrière A., Goulitquer S., Monsoor M., Duperier C. (2017). Create, run, share, publish, and reference your LC–MS, FIA–MS, GC–MS, and NMR data analysis workflows with the Workflow4Metabolomics 3.0 Galaxy online infrastructure for metabolomics. Int. J. Biochem. Cell Biol..

[46] Thévenot E.A., Roux A., Xu Y., Ezan E., Junot C. (2015). Analysis of the Human Adult Urinary Metabolome Variations with Age, Body Mass Index, and Gender by Implementing a Comprehensive Workflow for Univariate and OPLS Statistical Analyses. J. Proteome Res..

[47] Baba T., Campbell J.L., Le Blanc J.C.Y., Baker P.R. (2016). Structural identification of triacylglycerol isomers using electron impact excitation of ions from organics (EIEIO). J. Lipid Res..

[48] Nagy K., Sandoz L., Destaillats F., Schafer O. (2013). Mapping the regioisomeric distribution of fatty acids in triacylglycerols by hybrid mass spectrometry. J. Lipid Res..

[49] Bird S.S., Marur V.R., Sniatynski M.J., Greenberg H.K., Kristal B.S. (2011). Serum Lipidomics Profiling Using LC–MS and High-Energy Collisional Dissociation Fragmentation: Focus on Triglyceride Detection and Characterization. Anal. Chem..

[50] E Watkins L., Jones D.J., Mowrey D.H., Anderson D.B., Veenhuizen E.L. (1990). The effect of various levels of ractopamine hydrochloride on the performance and carcass characteristics of finishing swine. J. Anim. Sci..

[51] Council Directive 96/22/EC (1996). Council Directive 96/22/EC of 29 April 1996 Concerning the Prohibition on the Use in Stockfarming of Certain Substances Having a Hormonal or Thyrostatic Action and of Beta-Agonists, and Repealing Directives 81/602/EEC, 88/146/EEC and 88/299/EEC. http://data.europa.eu/eli/dir/1996/22/oj.

[52] Dunshea F.R. (1993). Effect of metabolism modifiers on lipid metabolism in the pig. J. Anim. Sci..

[53] Dunshea F.R., Leur B.J., Tilbrook A.J., King R.H. (1998). Ractopamine increases glucose turnover without affecting lipogenesis in the pig. Aust. J. Agric. Res..

[54] Ferreira M.S.D.S., Garbossa C.A.P., Oberlender G., Pereira L.J., Zangeronimo M.G., De Sousa R.V., Cantarelli V.D.S. (2013). Effect of ractopamine on lipid metabolism in vivo—A systematic review. Braz. Arch. Biol. Technol..

[55] Paris A., André F., Antignac J.P., Bonneau M., Briant C., Caraty A., Chilliard Y., Cognié Y., Combarnous Y., Cravedi J.P. (2008). L’utilisation des Hormones en Elevage: Les Développements Zootechniques et les Préoccupations de Santé Publique. https://hal.archives-ouvertes.fr/hal-01173447.

[56] Gotoh N., Moroda K., Watanabe H., Yoshinaga K., Tanaka M., Mizobe H., Ichioka K., Tokairin S., Wada S. (2008). Metabolism of odd-numbered fatty acids and even-numbered fatty acids in mouse. J. Oleo Sci..

[57] Collins J.R., Edwards B.R., Fredricks H.F., Van Mooy B.A.S. (2016). LOBSTAHS: An Adduct-Based Lipidomics Strategy for Discovery and Identification of Oxidative Stress Biomarkers. Anal. Chem..

[58] Koelmel J.P., Kroeger N.M., Ulmer C.Z., Bowden J.A., Patterson R.E., Cochran J.A., Beecher C.W.W., Garrett T.J., Yost R.A. (2017). LipidMatch: An automated workflow for rule-based lipid identification using untargeted high-resolution tandem mass spectrometry data. BMC Bioinform..

[59] Shen H., Huang J.Z. (2008). Sparse principal component analysis via regularized low rank matrix approximation. J. Multivar. Anal..

[60] Cao K.-A.L., Boitard S., Besse P. (2011). Sparse PLS discriminant analysis: Biologically relevant feature selection and graphical displays for multiclass problems. BMC Bioinform..

[61] Rinaudo P., Boudah S., Junot C., Thévenot E.A. (2016). biosigner: A New Method for the Discovery of Significant Molecular Signatures from Omics Data. Front. Mol. Biosci..

